# A versatile nanoreactor for complementary *in situ* X-ray and electron microscopy studies in catalysis and materials science

**DOI:** 10.1107/S160057751900660X

**Published:** 2019-08-27

**Authors:** Yakub Fam, Thomas L. Sheppard, Johannes Becher, Dennis Scherhaufer, Heinz Lambach, Satishkumar Kulkarni, Thomas F. Keller, Arne Wittstock, Felix Wittwer, Martin Seyrich, Dennis Brueckner, Maik Kahnt, Xiaogang Yang, Andreas Schropp, Andreas Stierle, Christian G. Schroer, Jan-Dierk Grunwaldt

**Affiliations:** a Institute for Chemical Technology and Polymer Chemistry, Karlsruhe Institute of Technology, Engesserstraße 20, Karlsruhe, Baden Württemberg 76131, Germany; b Institute of Catalysis Research and Technology, Karlsruhe Institute of Technology, Hermann-von-Helmholtz Platz 1, Eggenstein-Leopoldshafen, Baden Württemberg 76344, Germany; c Institute for Micro Process Engineering, Karlsruhe Institute of Technology, Hermann-von-Helmholtz Platz 1, Eggenstein-Leopoldshafen, Baden Württemberg 76344, Germany; d Deutsches Elektronen-Synchrotron DESY, Notkestraße 85, Hamburg 22607, Germany; e Department Physik, Universität Hamburg, Luruper Chaussee 149, Hamburg 22761, Germany; f Institute of Applied and Physical Chemistry, University of Bremen, Bremen 28359, Germany; g Faculty of Chemistry and Biochemistry, Ruhr-University Bochum, Universitätsstraße 150, Bochum 44801, Germany

**Keywords:** porous, nanoreactors, catalysis, X-ray microscopy, electron microscopy, hard X-ray ptychography

## Abstract

Two *in situ* ‘nanoreactors’ for high-resolution imaging of catalysts have been designed and applied at the hard X-ray nanoprobe endstation at beamline P06 of the PETRA III synchrotron radiation source. The reactors house samples supported on commercial MEMS chips, and were applied for complementary hard X-ray ptychography (23 nm spatial resolution) and transmission electron microscopy, with additional X-ray fluorescence measurements. The reactors allow pressures of 100 kPa and temperatures of up to 1573 K, offering a wide range of conditions relevant for catalysis. Ptychographic tomography was demonstrated at limited tilting angles of at least ±35° within the reactors and ±65° on the naked sample holders. Two case studies were selected to demonstrate the functionality of the reactors: (i) annealing of hierarchical nanoporous gold up to 923 K under inert He environment and (ii) acquisition of a ptychographic projection series at ±35° of a hierarchically structured macroporous zeolite sample under ambient conditions. The reactors are shown to be a flexible and modular platform for *in situ* studies in catalysis and materials science which may be adapted for a range of sample and experiment types, opening new characterization pathways in correlative multimodal *in situ* analysis of functional materials at work. The cells will presently be made available for all interested users of beamline P06 at PETRA III.

## Abstract 2

A series of nanoreactors have been developed for complementary *in situ* X-ray and electron microscopy. The setup was utilized to monitor thermal annealing processes of porous materials under controlled atmospheres with hard X-ray ptychography.

## 1. Introduction   

In the study of heterogeneous catalysis, derivation of structure–activity relationships is critical to unravel the role of catalysts in chemical processes as well as to improve their performance and stability. Since catalyst structure evolves with the chemical environment present, studies under working conditions (*in situ*) and with simultaneous acquisition of catalytic activity data (*operando*) are of high importance (Topsøe, 2003 ▸; Bañares, 2005 ▸; Grunwaldt & Clausen, 2002 ▸; Weckhuysen, 2002 ▸). As catalysts often possess a complex structure, another cornerstone in catalysis research is the collection of spatially resolved or local structural information. This is often achieved using imaging techniques such as electron or X-ray microscopy or a combination of these (Beale *et al.*, 2010 ▸; Frenkel & Van Bokhoven, 2014 ▸), especially if information at different length scales is required for a comprehensive understanding of the catalyst (Weckhuysen, 2009 ▸; Beale *et al.*, 2010 ▸; Grunwaldt *et al.*, 2013 ▸). Although superior spatial resolution is normally obtained by electron microscopy [*e.g.* sub-nm for transmission electron microscopy (TEM) and 1–50 nm for scanning electron microscopy (SEM)] and soft X-ray microscopy (de Smit *et al.*, 2008 ▸; Thomas & Hernandez-Garrido, 2009 ▸) for *in situ* measurements on real catalysts (*e.g.* powders, pellets), hard X-rays may be regarded as the more suitable probe. Hard X-rays may be applied on up to micrometre- to millimetre-sized samples under more practical working conditions, *e.g.* 100 kPa or elevated pressures, compared with soft X-ray or electron microscopy (Grunwaldt & Schroer, 2010 ▸; Buurmans & Weckhuysen, 2012 ▸). At the same time, development of hard X-ray focusing optics is constantly progressing towards smaller beam-spot sizes, and spatial resolutions comparable to those of soft X-ray beamlines (Schroer *et al.*, 2017 ▸; Seiboth *et al.*, 2017 ▸; da Silva *et al.*, 2017 ▸; Huang *et al.*, 2015 ▸; Yan *et al.*, 2014 ▸). In terms of spatial resolution, scanning coherent X-ray diffraction imaging, also known as X-ray ptychography (XRP), has been the main X-ray imaging technique to make a significant breakthrough, *i.e.* 5 nm for soft X-rays (Shapiro *et al.*, 2014 ▸) and 10 nm for hard X-rays (Vila-Comamala *et al.*, 2011 ▸; Schropp *et al.*, 2012 ▸). Ptychography employs a coherent X-ray beam to scan over the sample, while recording far-field diffraction patterns with a partial overlap between adjacent points in real space. By using iterative phase-retrieval algorithms, the overlapping diffraction patterns are then reconstructed into real-space images. The use of highly coherent flux and the absence of focusing optics between the sample and detector effectively means that the spatial resolution of ptychography is not limited by the beam-spot size, offering excellent potential for high-resolution imaging (Guizar-Sicairos *et al.*, 2015 ▸; Schropp *et al.*, 2012 ▸). However, combining *in situ* measurements with spatially resolved microscopy at the nanometre scale, including complementary characterization of identical samples by multiple techniques, is a challenging but highly valuable prospect (Weker *et al.*, 2016 ▸). While XRP is often used *ex situ* for many applications (Thibault *et al.*, 2008 ▸; Hoppe *et al.*, 2013 ▸; Piazza *et al.*, 2014 ▸; Zhu *et al.*, 2015 ▸; Wise *et al.*, 2016 ▸; Sala *et al.*, 2018 ▸), *in situ* imaging modes, particularly on catalyst materials where accurate control of temperature and gas conditions is essential, have not been widely explored outside of a few pioneering works (Høydalsvik *et al.*, 2014 ▸; van Riessen *et al.*, 2017 ▸), including those from our group (Baier, Damsgaard *et al.*, 2016 ▸; Baier, Wittstock *et al.*, 2016 ▸; Baier *et al.*, 2017 ▸). Nevertheless, these studies still leave room for improvement in terms of spatial resolution, detection of possible reaction products and the application of complementary imaging modes [*e.g.* TEM and X-ray fluorescence (XRF)], as discussed below. An outlook on the experimental potential of *in situ* X-ray imaging by a range of methods including XRP was recently outlined in the literature (Weker *et al.*, 2016 ▸). Like other X-ray imaging methods, ptychography can also be applied for tomographic analysis of interior sample structures through a technique known as ptychographic X-ray computed tomography (PXCT) (Dierolf *et al.*, 2010 ▸). In catalysis research, PXCT has recently been exploited, for example, to observe and label pore networks (Li *et al.*, 2019 ▸; da Silva *et al.*, 2015 ▸), or to identify chemical constituents and their location within global catalyst particles (Ihli *et al.*, 2017 ▸, 2018 ▸), while 3D spatial resolutions approaching 23 nm have been demonstrated (Fam *et al.*, 2018 ▸). However, since the experimental demands of tomography are significantly more complex than for 2D imaging, performing PXCT *in situ* under precisely controlled gas and temperature conditions undoubtedly constitutes an even greater challenge than for *in situ* ptychography.

One of the first *in situ* XRP studies on catalytic materials was carried out to probe lithium zirconate for CO_2_ capture applications. A spatial resolution of 200 nm (Høydalsvik *et al.*, 2014 ▸) was achieved, although the experiment was performed in a large gas-filled enclosure rather than a closed *in situ* cell with controlled internal volume and gas atmosphere. Simultaneous use of *in situ* XRP and XRF nanospectroscopy was later shown to monitor morphological changes and elemental distribution during thermal curing of geopolymers on sub-micrometre length scales, though the experimental apparatus was not fully discussed (van Riessen *et al.*, 2017 ▸). We previously described the design and application of closed *in situ* cells based on micro-electro-mechanical systems (MEMS) chips for XRP of catalyst materials, and studied morphological changes of colloidal gold nanoparticles and nanoporous gold catalysts during thermal annealing under specific gas atmospheres with spatial resolutions down to 20 nm (Baier, Damsgaard *et al.*, 2016 ▸; Baier, Wittstock *et al.*, 2016 ▸). Another application was to study the stability of a CuO/ZnO/Al_2_O_3_-ZSM-5 core-shell material under reducing and oxidizing conditions, obtaining 30 nm spatial resolution (Baier *et al.*, 2017 ▸). Notably, the previous *in situ* cells could also be used for complementary electron microscopy on identical sample regions. However, the aforementioned setup (Baier, Damsgaard *et al.*, 2016 ▸) possessed some limitations. Apart from a relatively bulky design, high mass (>100 g) and large internal volume, it was not possible to perform complementary XRF imaging or quantify products for *operando* studies, while sample heating was monitored and controlled indirectly via infrared (IR) thermography. Hence, further improvements are required particularly with regard to sample stability, mechanical vibrations and experimental control of temperature. Recently, an alternative cell based on MEMS chips was reported for 2D scanning transmission X-ray microscopy (STXM) of Fischer–Tropsch synthesis catalysts under *in situ* conditions (van Ravenhorst *et al.*, 2018 ▸). In this case, the cell was applied using soft X-rays up to around 800 eV, which necessitates the preparation of thin samples in comparison with hard X-ray ptychography.

In this study, we present a series of new and improved *in situ* cells based on MEMS chips for complementary X-ray nano­imaging and spectroscopy measurements of catalysts and other functional materials using synchrotron radiation. The cells were designed to allow high-resolution imaging in 2D (2G cell) and tomographic imaging in 3D (3G cell) with limited rotational angles, where 3D volume data may be reconstructed via missing-wedge tomography algorithms without the need to perform a full 180° rotation of the sample. In both cases, the cells can operate under *in situ* conditions, *i.e.* with controlled temperature up to 1573 K and gas environments up to 100 kPa. Firstly, the design principles and main features of the 2G and 3G cells are introduced, including discussion on the improvement from previous cell designs. Next, experimental case studies are introduced with the newly commissioned cells. 2D XRP was used to monitor thermal sintering and annealing of monolithic nanoporous gold (np-Au) as a bulk catalyst material. A related example of complementary XRP and XRF mapping of 50 nm gold colloids during sintering is shown in the supporting information. Furthermore, to demonstrate the possibility of performing 3D PXCT measurements in the cells, a hierarchical micro/macroporous zeolite particle was investigated under a limited rotational angle series of ±35°, with the possibility to extend this to ±65° depending on experimental needs.

## 2. Experimental   

### 2.1. Design of the *in situ* cells and experimental setup   

The *in situ* cells used in this study were designed specifically for use at the hard X-ray nanoprobe endstation at beamline P06 at the PETRA III synchrotron radiation source (Hamburg, Germany), although, in principle, the design is flexible enough for incorporation at similar nanoprobe beamlines. Two *in situ* cells with specific target functions were commissioned: (i) an *in situ* cell for high-resolution *in situ* XRP in 2D, with complementary XRF and/or large-angle X-ray diffraction (XRD) mapping, denoted as ‘2G cell’; and (ii) an *in situ* cell allowing limited-angle 3D *in situ* PXCT in addition to standard 2D XRP, denoted as ‘3G cell’.

**Figure 1:**
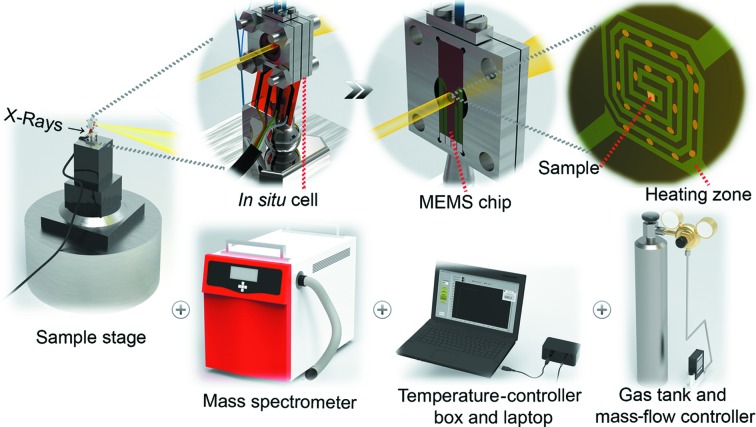
Illustration of the *in situ* setup for 2D and 3D ptychography at the P06 nanoprobe endstation of PETRA III. Zooming into the *in situ* cell (top), and gas and temperature control infrastructure and product analytics (bottom).

Both cells are based on modular design principles and can be interchangeably integrated into the same setup (Fig. 1 ▸). For accurate temperature control, a Digiheater temperature-controller box and software are used (DENSsolutions, Delft, The Netherlands), with printed electrical contacts to interface with the MEMS chip. Mass-flow controllers (Bronkhorst High-Tech BV, Ruurlo, The Netherlands) provide accurate control of the gas environment, while a mass spectrometer (Thermostar, Pfeiffer Vacuum GmbH, Aßlar, Germany) positioned at the reactor outlet allows for gas analysis. The cell is fixed in a standing position on the beamline stage (Fig. 1 ▸, top left), which includes lateral piezomotors for nanopositioning and a goniometer for sample rotation. Within the cell, there is a Wildfire MEMS chip (DENSsolutions, Delft, The Netherlands) on which samples are deposited on the center spiral-shaped sample area (Fig. 1 ▸, top right). The sample area may be with or without silicon nitride (Si_3_N_4_) membranes, depending on the experimental requirements and the type of chip used. The operational range of the Wildfire chip is certified at 100 kPa pressure and up to 1373 K with Si_3_N_4_ membranes, or 1573 K with through-hole chips. The heating zone is indicated by the spiral but it has been proven to be well localized within 0.5 mm from the center point. Since the MEMS chips have been commercially utilized for TEM, complementary TEM measurements may readily be performed along with *in situ* XRP and PXCT by simply removing the chip and placing it in an appropriate electron-microscopy sample holder.

**Figure 2:**
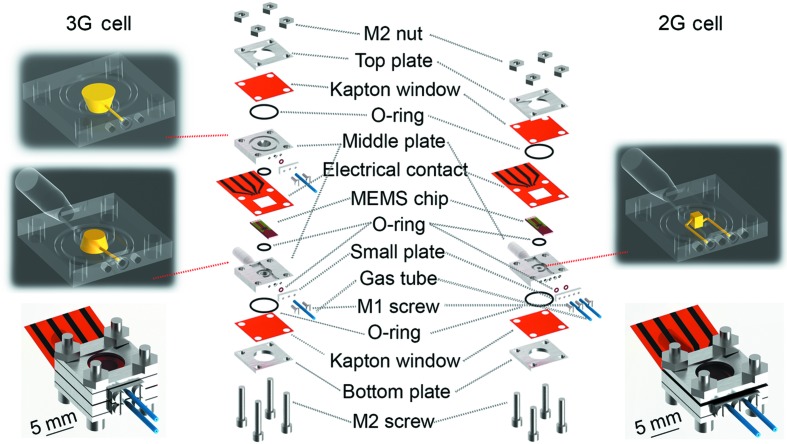
Illustration of the disassembled 2G (right) and 3G (left) cell with their internal gas-flow volumes (highlighted above). The fully assembled setups are shown below.

Both the 2G and 3G cells have the same general configuration, where the top and bottom plates containing the MEMS chip are fixed between O-rings and Kapton films (Fig. 2 ▸). Small side plates and O-rings provide gas-tight sealing of the inlet/outlet gas tubes. Both cells have a similar arrangement for the electrical contact, in the form of a printed circuit (Au on Kapton) which should be positioned on top of the MEMS chip so that the four contact pads on each are well aligned. This electrical contact serves to deliver the current from the temperature-controller box (power supply) to the MEMS chip, such that the chip can convert the electrical supply into heat based on the Joule-heating principle. The final similarity between the 2G and 3G cells comes from the small O-ring below the MEMS chip, which also provides gas-tight operating conditions for the cell.

**Figure 3:**
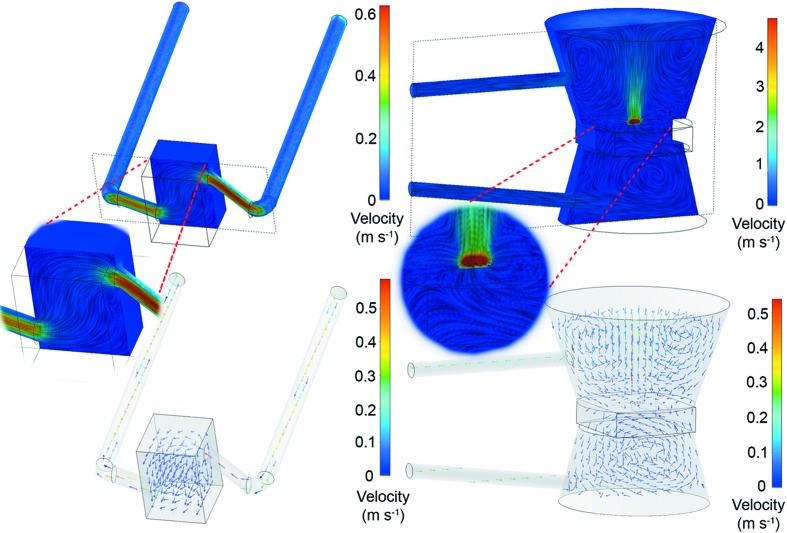
Internal gas-flow volume and simulation of the 2G cell (left) and the 3G cell (right).

Beyond these aspects, there are some noticeable differences in design and function between the 2G and 3G cells. The use of one middle plate for 2G along with Si_3_N_4_ membrane MEMS chips, but two middle plates for 3G with through-hole MEMS chips, directly influences the internal volume of the cells. The 2G cell has around 2.6 µL internal volume and the 3G cell has around 24.5 µL, thus the word ‘nano­reactor’ is used in this work because of the internal volumes approaching 1 µL. Fluid-flow simulations were performed to demonstrate the gas-flow principle in each cell as shown in Fig. 3 ▸ (description in Section S1 of the supporting information). For 2G, the gas flows into the single middle plate, across the underside of the sample deposited on the back of MEMS chip, and then out once again from the same plate (Fig. 3 ▸). Meanwhile for 3G, the gas flows from the bottom middle plate, directly through the sample deposited on the through-hole MEMS chip, and finally to the outlet port of the top middle plate (Fig. 3 ▸). Sample drift as a result of gas flow/pressure in the cell is expected to be negligible, because of a low Reynold’s number of 9, while the MEMS chips are typically used for environmental TEM which is considerably more sensitive to sample drift because of the highly focused electron beam. Regarding cell mounting, the 2G cell has to be placed directly perpendicular to the incident X-ray beam, while the 3G cell allows tilting of ±35° around the center of rotation with respect to the beam (or ±65° for the naked MEMS chip). The intention is that all kind of samples can be used for 2G, including colloids, size-selected clusters or unsupported samples which require a membrane for stability. However, imaging studies are then limited to 2D XRP. On the other hand, the 3G cell with through-hole chips can exploit both 2D XRP and limited-angle PXCT, but this is restricted to pre-shaped samples which are then fixed to the chip through focused ion beam (FIB) manipulation or similar techniques. The general intention is to provide maximum flexibility to users of the hard X-ray nanoprobe endstation of beamline P06, who often have diverse sample requirements and structural features of interest.

### 2.2. Sample preparation and mounting   

In terms of the sample dimensions, in the plane of the MEMS chip (*x* and *y* axis) they can reach up to 0.5 mm × 0.5 mm, equivalent to the heating area. However, the maximum sample thickness (*z* axis, vertical height above the heating area) has not been definitively shown. While the cell can physically accommodate samples of several hundred micrometres thickness, to obtain uniform heating the sample should probably not extend more than several tens of micrometres above the heating area. Ensuring uniform heating will also greatly depend on the sample, with respect to thermal conductivity. Since technical catalysts often possess dimensions up to the millimetre scale, an accurate *in situ* experiment will typically involve a degree of sample preparation. For example, this may be through selection of small grains of a catalyst sample through abrasion, grinding or sieving from larger bodies, analogous to TEM sample preparation. Alternatively, for extracting sub-volumes of shaped or hierarchically structured materials where preservation of the structure is essential, the use of FIB, He-ion microscopes or similar instruments may be required. This is necessary both to shape the sample and to deposit it correctly on the MEMS chips. Preparation of samples representative of the global material of interest should therefore be carefully considered. However, it should be noted that the range of typical sample dimensions permitted still greatly exceeds those feasible using TEM, because of relatively greater attenuation of electrons compared with hard X-rays.

**Figure 4:**
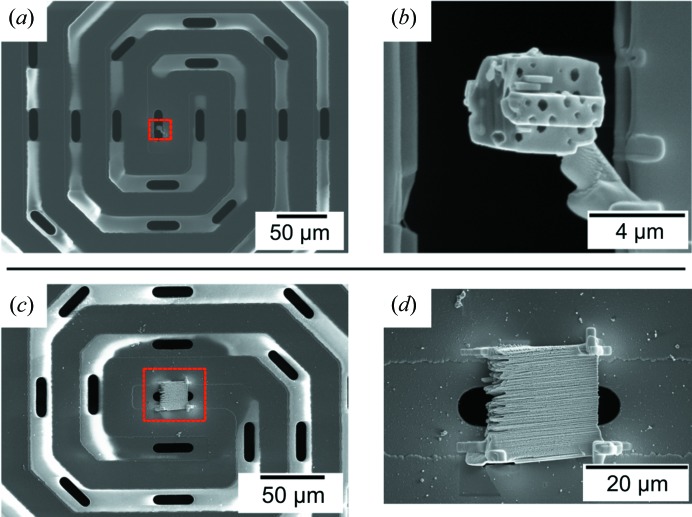
SEM images obtained during FIB preparation on Wildfire chips at DESY NanoLab: (*a*) micro/macroporous zeolite crystal and (*b*) zoom-in of the highlighted area; (*c*) monolithic np-Au wedge and (*d*) zoom-in of the highlighted area.

Two sample types were used to demonstrate the use of the cells: (i) monolithic np-Au and (ii) hierarchical zeolite with intracrystalline macropores. Pure np-Au samples were prepared by dealloying of an Ag–Au alloy (*i.e.* 70 at% Ag and 30 at% Au) in nitric acid (65 wt%), as previously published (Lackmann *et al.*, 2018 ▸). The sample was placed on an SEM sample-holder stub (Fig. S1), introduced to the FIB setup, and then cut and shaped with a Ga^+^ ion beam into a wedge (Figs. S2 and S3). The thicker part (1 µm) was used for *in situ* XRP and the thinner part (0.2 µm) was used for *ex situ* TEM. The wedge was extracted with the micromanipulator and placed across the window of a through-hole Wildfire chip (Fig. S4), before being fixed by Pt glue at each corner. The as-prepared np-Au wedge sample is shown in Figs. 4 ▸(*c*) and 4 ▸(*d*). Macroporous zeolites were synthesized by a templating method using silica and alumina precursors, followed by steam-assisted crystallization to form macropores within the crystals, as described in previous publications (Machoke *et al.*, 2015 ▸; Schwieger *et al.*, 2016 ▸). The zeolite powder consisting of 2–4 µm crystals was introduced to the FIB setup, with a suitable grain selected and placed across the window of a through-hole Wildfire chip, before being fixed with Pt glue on one side. The as-prepared zeolite sample is shown in Figs. 4 ▸(*a*) and 4 ▸(*b*). Sample preparation by FIB was performed at DESY NanoLab (Stierle *et al.*, 2016 ▸). A detailed description of the different sample-preparation steps is illustrated in the supporting information (Figs. S1–S4). TEM, energy-dispersive X-ray spectroscopy (EDX) and selected-area electron diffraction (SAED) measurement were performed using a Wildfire sample holder (DENSsolutions, Delft, The Netherlands), on a Titan 80-300 (FEI) microscope operated at an acceleration voltage of 300 kV in STEM and TEM mode, at the Institute of Nanotechnology (INT) at Karlsruhe Institute of Technology.

### 2.3. *In situ* synchrotron X-ray imaging studies   

All experiments were performed at the nanofocus endstation of beamline P06 at the PETRA III synchrotron radiation source (Hamburg, Germany) using the measurement parameters shown in Table 1 ▸. All XRP measurements were performed using an EIGER X 4M detector (DECTRIS Ltd, Switzerland) with 75 µm × 75 µm pixel size and 2070 × 2167 pixels. Measurement of np-Au and macroporous zeolites was performed during two separate beam times. For measuring the whole sample of np-Au, a field of view of 20 µm × 20 µm was used with 14 min scan time per image. For the smaller 5 µm × 5 µm field of view indicated in Table 1 ▸, a region of interest was selected from the center of the sample wedge. The different choice of exposure time for the np-Au and zeolite samples is due to measurement in the focal position with high scattering (pure Au sample), compared with measuring out of focus on a relatively weaker scattering sample (zeolite, Si/Al).

For *in situ* measurement of the np-Au sample, pure helium with a specific inlet flow of 1 ml min^−1^ adjusted by a mass-flow controller was used to maintain a controlled atmosphere during *in situ* annealing treatment. The temperature was controlled via Joule heating and monitored using customized Labview software (National Instruments, USA). Specific temperature conditions applied during imaging are indicated next to the relevant figures in the results section[Sec sec3]. The outlet flow from the cell was analyzed online using a Thermostar quadrupole mass spectrometer (Pfeiffer Vacuum GmbH, Aßlar, Germany) to confirm that the desired atmospheric composition was present during *in situ* experiments. In this case, He and residual O_2_ (*m*/*z* = 4 and 32, respectively) were monitored for the inert atmosphere. For measurement of the hierarchical zeolite by limited-angle tomography, the sample on a Wildfire chip was placed within the 3G cell and aligned to the center of rotation with respect to the beam. 2D ptychographic projections were recorded across an angular range of approximately ±35° in 1.4° steps, leading to a total of 51 projections. The zeolite was scanned in a raster grid with each scan point randomly offset in both scanning directions by up to 50% of the step size, known as grid jitter mode.

### 2.4. Data processing   

The algorithm used for ptychographic reconstruction was based on the ePIE algorithm (Maiden & Rodenburg, 2009 ▸). Cropping the diffraction patterns to 256 × 256 pixels led to a pixel size of 8.2 nm (for np-Au), and 8.5 nm (for zeolite) in the reconstructed images. To estimate the spatial resolution, a Fourier ring correlation analysis (van Heel & Schatz, 2005 ▸) was performed (Figs. S6–S8). Correlation was always performed on 2D projections (ring correlation), as opposed to 3D volumes (shell correlation). As a common procedure, two individual successive scans of an identical sample area were reconstructed and compared. First, the images were cropped to exclude the edges of the field of view, phase wedges were removed and the images were aligned on a sub-pixel level using the *SciPy* Python package (Guizar-Sicairos *et al.*, 2008 ▸). Before correlating the phase reconstruction, a Kaiser–Bessel window function with a size equal to 1 was applied to the images, in order to reduce artifacts caused by erroneous high frequencies resulting from the edges of the limited field of view of the reconstructions.

Tomography reconstruction of the ptychographic projection series for the hierarchical zeolite sample was performed as follows. Firstly, the 51 reconstructed phase projections were cropped to only include the sample area present within the window of the MEMS chip. Secondly, phase wedges and offsets were removed from the projections, using the regions right next to the sample as the zero-reference area. The third step was to align all projections to each other in the vertical direction (along the rotation axis) by correlating the derivative of their vertical profiles (Guizar-Sicairos *et al.*, 2011 ▸). Step four was the horizontal alignment (in the tomographic plane) by aligning the horizontal center-of-mass of all the projections. This placed the rotation axis into the center-of-mass of the particle. The obtained sinogram was reconstructed using multiple tomography algorithms, including: (i) the simultaneous iterative reconstruction technique (SIRT) from the *TomoJ* package (Messaoudii *et al.*, 2007 ▸), as implemented in the *FIJI* software (Schindelin *et al.*, 2012 ▸); (ii) the simultaneous algebraic reconstruction technique (SART) (Andersen & Kak, 1984 ▸), as part of the Python package *scikit-image* (van der Walt *et al.*, 2014 ▸); (iii) the maximum-likelihood expectation maximization (MLEM) algorithm using in-house code based on the literature (Bruyant, 2002 ▸); and (iv) an in-house deep-learning code in development at PETRA III for analysis of limited-angle tomography data (in preparation).

## 3. Results   

### 3.1. Design process for the 2G and 3G *in situ* cells   

The design of the 2G and 3G cells was directly inspired by the first-generation *in situ* XRP cell developed by our group, denoted here as the 1G cell (Baier, Damsgaard *et al.*, 2016 ▸). Development of the next-generation cells was driven by several significant practical limitations of our own 1G cell and those previously described in the literature, which are summarized in the following section.

One of the first applications of *in situ* 2D XRP was to monitor CO_2_ capture on Li_2_ZrO_3_ materials, which was performed in a plexiglass sample enclosure. However, such an open sample environment is considerably different in volume to a closed reactor or cell. Therefore, both exclusion of ambient air and maintaining a defined gaseous environment such as CO_2_ are challenging tasks, particularly if haza­rdous gases are required (Høydalsvik *et al.*, 2014 ▸). Later experiments used a scanning chip calorimeter to visualize thermal annealing processes in organic photovoltaic layers in combination with 2D XRP (Patil *et al.*, 2016 ▸; Van den Brande *et al.*, 2017 ▸). Although showing an excellent control over annealing temperatures with this setup, the same issue with controlling the gas environment is present and such a system is not optimal for heterogeneous catalysis studies with defined reaction conditions. A separate study used *in situ* XRP to observe the microstructure changes of geopolymers during thermal curing processes (van Riessen *et al.*, 2017 ▸), but unfortunately the setup and sample environment were not detailed, while it appears that the samples were heated in an open atmosphere. Recently an alternative cell design based on MEMS chips was reported with some similarities to that of the current work (van Ravenhorst *et al.*, 2018 ▸). The authors demonstrated the application of STXM on a Fischer–Tropsch catalyst particle with 50 nm spatial resolution, specifically by performing spectroscopic measurements with soft X-rays around the Co *L*_2–3_, Ti *L*_2–3_ and C *K* edges in absorption-contrast mode. However, soft X-rays generally necessitate more invasive preparation of thin samples compared with hard X-rays. Furthermore, the geometry of the cell (*e.g.* beamline mounting, window-exit angle) may not permit complementary measurements such as nanofluorescence or nanodiffraction, limiting the experimental flexibility of the system compared with the 2G/3G cell setup described here. Furthermore, the highly focused 50 nm beam for STXM requires strict minimization of sample drift and mechanical vibrations. On the other hand, ptychography can offer superior spatial resolution independent of the beam-spot size, allowing samples to be scanned in or out of the beam focal point (Schropp *et al.*, 2012 ▸; Guizar-Sicairos *et al.*, 2015 ▸; Hönig *et al.*, 2011 ▸), as shown in the current work. Measuring out of focus can generally result in more rapid scan times over larger sample volumes for ptychography while relaxing the limitations of sample drift over time, compared with STXM which requires a highly focused beam to obtain high spatial resolution. Furthermore, XRP offers the possibility to obtain complementary spectroscopic information through resonant XRP imaging (Hoppe *et al.*, 2013 ▸; Ihli *et al.*, 2018 ▸), or to identify and locate sample constituents by converting the observed phase shift to yield electron-density maps of the sample (Diaz *et al.*, 2012 ▸). Unlike the 3G cell, the geometry of the alternative MEMS chip design is also not suitable for 3D imaging either in electron microscopy or potentially with PXCT, although complementary TEM studies would be feasible.

Our previous work with the 1G cell was able to demonstrate complementary TEM and XRM, a contained gas environment and a wide range of applicable temperatures with indirect heating provided by a variable voltage power supply and an IR thermography camera. A point-by-point comparison of the 1G cell with the 2G and 3G cells presented in this work follows. The 2G and 3G cells described here have been improved with respect to many of the criteria mentioned above, particularly with respect to the 1G cell developed by our group. Firstly, they both exhibit significantly smaller internal gas-flow volumes (volume ratio of 1G:2G:3G = 500:2.6:24.5 µL = 192:1:9). This is an important factor in determining the sample sizes necessary for *operando* studies, where the smaller the internal volumes, the more freedom available to use smaller sample sizes while being able to reliably detect dilute products. On the other hand, where relatively larger sample volumes are used, reactant conversion may be high enough to allow quantitative product analysis in addition to imaging. Therefore, the sample size is an important criterion for the purpose of mass-spectrometry characterization of the gas-stream outlet, but it is also relevant if complementary TEM studies are required. Preparation of relatively large or thick samples by FIB is time intensive and may result in attenuation of the electron beam and poor resolution. The ability to effectively image either thick samples with X-ray imaging, or thin samples with electron and X-ray imaging adds a layer of flexibility to the possible uses of the setup (see the np-Au wedge study below). In addition, the low internal cell volume actually leads to a smaller physical profile of the cell, which generally decreases the total weight of the setup. This is crucial to ensure accurate and error-free nano-precise positioning of the setup by the beamline sample stage motors. The 2G and 3G cells therefore come with a significant decrease in size and mass compared with the 1G cell and other cells reported earlier.

Another advantage of the current cells is the additional characterization capabilities on offer. Spectroscopic XRF analysis is possible in addition to XRP, which is extremely useful to map the elemental distribution within the probed material. Although methods are available to accurately determine elemental speciation using XRP [*e.g.* resonant imaging around an elemental absorption edge (Hoppe *et al.*, 2013 ▸)], XRP does not always guarantee chemical contrast in the sample of interest. For ptychography beamlines optimized in the use of nanofocusing optics, such as P06 at PETRA III, the acquisition of highly spatially resolved local fluorescence data is a natural advantage because of the small beam size. Provided the incident energy is above the absorption edge of interest, XRF mapping is complementary to XRP. Such measurements were not possible with the 1G cell because of its bulky cell body, which did not allow detector positioning at an appropriate angle to detect a measurable fluorescence signal. At the moment, XRF has to be performed following slight rotation (25–35°) of the cells to face the detector, which can be positioned around 110° relative to the incident X-ray rather than the optimal angle of 90°. This also means that simultaneous XRF and XRP are not optimal, although they can be sequentially acquired on congruent sample fields of view with little delay between measurements. However, the necessary beam conditions for simultaneous XRF and XRP measurement modes are not always complementary in every case. Furthermore, the 3G cell has been designed for PXCT with limited angles, similar to the missing-wedge principle commonly employed in electron tomography. This allows for large-angle XRD up to a 2θ range of around 70°. On the contrary, the 1G cell was strictly limited to 2D XRP.

The temperature-control system is one of the most significant improvements over the original 1G setup. The 2G and 3G cells exhibit a direct and fast response to programmed temperature input because of the use of MEMS chips with 4-point resistivity measurement, allowing temperature readout and control in a feedback loop (see Fig. S5). This capability has also been widely used in the *in situ* Wildfire series TEM sample holders (Ren *et al.*, 2018 ▸; Gocyla *et al.*, 2018 ▸). Furthermore, the specific resistivity of each chip under ambient conditions is calibrated during manufacture, improving the accuracy of all temperature-controlled experiments. With the 1G cell, no temperature feedback was used, necessitating a variable voltage power supply system and indirect temperature measurements with an IR camera, whose temperature readout and control is significantly less effective. This is partly because of the delay in recording the temperature from the camera and manually adjusting the power supply to obtain the desired condition. However, the indirect control mechanism also leaves the system vulnerable to unexpected changes in temperature, *e.g.* from changing gas conditions and therefore specific heat capacity of the local environment, or from thermal variation because of chemical reactions taking place on the chip. In summary, the cells offer significant current and potential improvements over similar setups previously described in the literature. For example, (i) compatibility with a range of sample types, including colloids, metal nanoparticles, bulk materials and single catalyst grains. (ii) Maintenance of a closed cell with an easily controllable gas environment, allowing for the application of oxidative, reductive or haza­rdous gases in a safe manner. (iii) Small ratio of internal cell volume to sample volume, permitting high contact time between samples and reactant gases, and potentially facilitating the detection of gaseous products during X-ray imaging when large enough samples are present (*operando* imaging). (iv) Low mass of the setup, mitigating vibrational instabilities and positioning errors when using nanoprecise sample stage motors, thereby leading to improved spatial resolution. (v) Capability to perform several complementary measurements (possibly simultaneously), including XRF and large-angle XRD (nanospectroscopy/nanodiffraction). (vi) Capability to perform complementary electron microscopy and ptychography on identical samples, including *in situ* electron microscopy with an appropriate sample holder. (vii) Capability to perform 3D imaging by acquiring 2D projections at limited rotational angles. (viii) Direct and accurate resistive heating, which may be easily programmed, monitored and precisely controlled via a feedback mechanism (±0.01 K in Fig. S5) under specific desired gas atmospheres (*e.g.* inert He, O_2_, H_2_, reactive gases).

### 3.2. Annealing of np-Au series in a controlled gas environment using the 2G cell   

**Figure 5:**
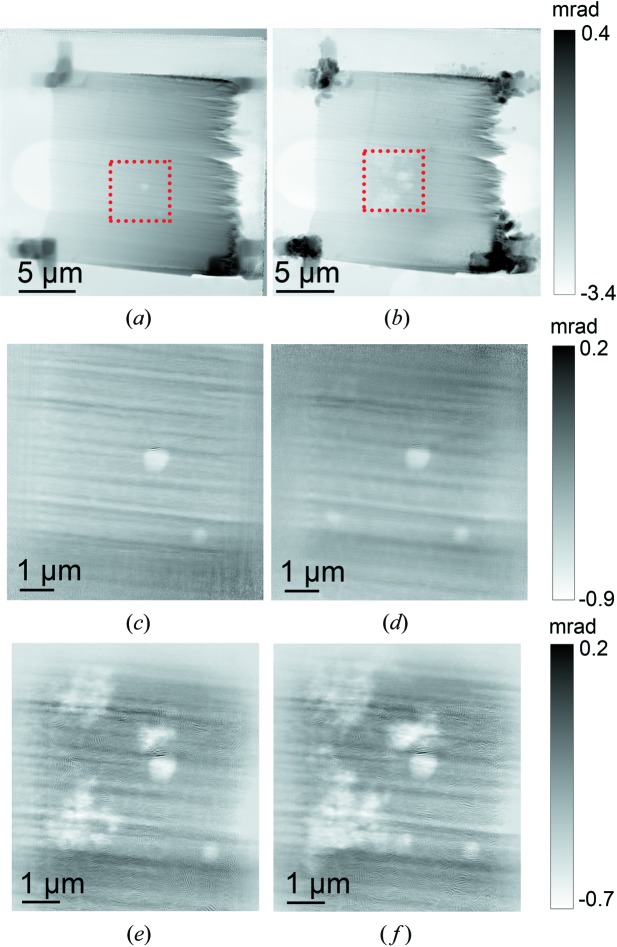
Ptychography images from thermal annealing of np-Au under a He environment. Overview scans: (*a*) before and (*b*) after thermal annealing. Highlighted area in (*a*) and (*b*) shown: (*c*) at 293 K, (*d*) 923 K after 0 min, (*e*) 923 K after 15 min and (*f*) 923 K after 30 min. Darker intensity in phase maps indicates areas rich in Au, while brighter voids indicate annealing and absence of Au ligaments. Pt glue from FIB is visible as intense features in the corners of (*a*) and (*b*).

The annealing of hierarchically structured np-Au was chosen as a case study to demonstrate the capabilities of the fully commissioned *in situ* cells (Fig. 2 ▸) and related infrastructure (Fig. 1 ▸). Nanoporous gold is an ideal sample for XRP because of its high phase contrast, and the large difference in electron density between the substrate and background air (Fam *et al.*, 2018 ▸). Moreover, np-Au has a hierarchical structure of a sponge-like 3D network, where its pores and ligaments form a macroscopic material spanning from nanometre to millimetre scales (Wichmann *et al.*, 2013 ▸; Bagge-Hansen *et al.*, 2014 ▸; Shi *et al.*, 2014 ▸, 2016 ▸, 2017 ▸). This makes np-Au an excellent benchmark for evaluating the highest resolution achievable under *in situ* conditions (Larsson *et al.*, 2019 ▸). Experiments were performed at elevated temperatures up to 923 K while flowing He (1 mL min^−1^) through the cell. A large 20 µm × 20 µm overview scan of the np-Au wedge was recorded before and after heat treatment. For the high-resolution *in situ* imaging series, a smaller 5 µm × 5 µm field of view was selected from the center of the sample wedge. XRP reconstructions of the overview scan and selected *in situ* images are shown in Fig. 5 ▸, with an estimated spatial resolution of 23 nm (Fig. S7). The through-hole window of the MEMS chip can be observed as a lighter rounded rectangle shape in the middle of Figs. 5 ▸(*a*) and 5 ▸(*b*). Notably for hard X-ray imaging it is not strictly necessary to place the sample directly over such a window, although this will reduce background scattering. On the other hand, for potential TEM measurements, the sample must be placed over such a window.

The sample was thermally stable under inert gas atmosphere up to 923 K, after which the coarsening process was strongly visible. The temperature was kept constant at 923 K and sequential 2D XRP images were acquired to illustrate the progression of the coarsening process. The high accuracy of the temperature-control system can be seen in the experimental readout plot (see Fig. S5). Coarsening was observed as large-scale annealing of fine gold ligaments (which were previously at or below the resolution limit), to form larger ligaments and leaving behind irregular-shaped voids of lower electron density, therefore lower local concentration of Au. Individual ligaments are faintly visible around the voids in Figs. 5 ▸(*e*) and 5 ▸(*f*). In comparison with previous experiments on np-Au, the coarsening process was studied by both *in situ* TEM and *in situ* ptychography, with the onset of coarsening found to be at 523–573 K in 100 kPa of synthetic air (20% O_2_/N_2_), 573 K in 320 Pa of pure O_2_ and 973 K in vacuum (Baier, Damsgaard *et al.*, 2016 ▸; Baier, Wittstock *et al.*, 2016 ▸). It was previously concluded that both the atmosphere composition and the pressure affect material stability. An oxygen-containing atmosphere at any pressure may reasonably be expected to interact more strongly with the Au surface than inert He, therefore the onset of coarsening observed here at 923 K lies somewhere between the values observed in synthetic air or pure oxygen, and vacuum conditions. This additionally proves the maintenance of an inert environment inside the cell. A notable improvement over previous measurements is that these were carried out at 100 K intervals (TEM studies) and 25–30 K intervals (*in situ* XRP), whereas here it was possible to use consecutive temperature steps of 10 K, leading to greater precision in determining the coarsening onset temperature. Comparing the first and last overview image in Figs. 5 ▸(*a*) and 5 ▸(*b*), it should be noted that the Pt glue fixed on the corner of the sample for stability via FIB partly disintegrated. For future studies, the deposition of more temperature stable materials, *e.g.* tungsten, may be an advantage. Another important point to consider in this case is the unavoidable thermal drift caused by expansion of the MEMS chip viewing area, which prompts users either to find a ‘natural’ marker for determining the extent of drift from the area of interest, or to make a synthetic marker, *e.g.* deposition of Pt ring, via FIB prior to XRP measurements. In this experiment, the edge of the MEMS chip window was used as a natural marker. Also notable is how the coarsening process appeared to be more intense precisely in the area scanned continually during the temperature series. This aggravated coarsening behavior is suggested to be influenced by beam effects, since the area outside the field of view was less affected.

**Figure 6:**
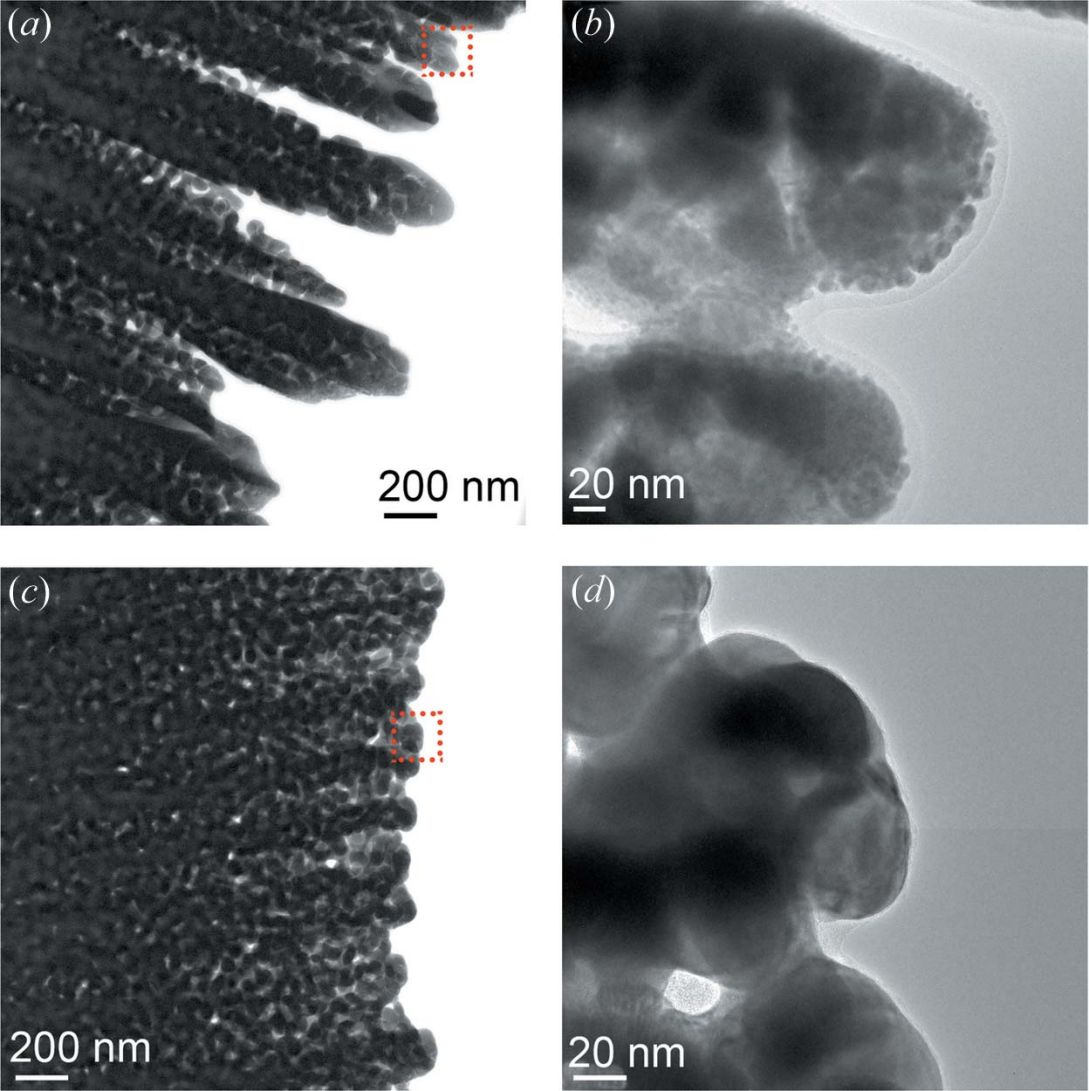
TEM images of np-Au: (*a*) before *in situ* thermal annealing and (*b*) a zoom-in on the highlighted area; (*c*) after *in situ* thermal annealing at 923 K and (*d*) a zoom-in on the highlighted area.

To demonstrate the possibility for complementary measurements on a single sample, TEM was conducted on the thin part of the np-Au wedge before and after *in situ* XRP, as shown in Fig. 6 ▸. The structural difference is noticeable particularly between Figs. 6 ▸(*b*)–6 ▸(*d*), where the np-Au ligaments appear more consolidated as expected following thermal annealing at 923 K (Baier, Damsgaard *et al.*, 2016 ▸; Baier, Wittstock *et al.*, 2016 ▸). In addition, some minor contamination was observed at the border of the ligaments [Fig. 6 ▸(*b*)], which was characterized as containing Si and Pt using EDX analysis (see Fig. S12 and EDX quantification in Table S1). Such contamination could originate from the Si_3_N_4_ layer present on the MEMS chips or the Si chip body, which may have been deposited during the process of attaching the sample wedge to the Wildfire chip by FIB. This would also explain the source of the Pt contamination during gluing of the wedge to the MEMS chip. The Si layer was still detected even after thermal treatment [Fig. 6 ▸(*d*)], although it became significantly thinner probably because of the influence of high temperature. While such contamination is unfortunate, it is also a common feature of FIB preparation and is difficult to exclude; however, it is not expected that the complete sample wedge was affected particularly for XRP measurements, where the sample thickness in the region of interest was estimated as 600 nm. The composition of the np-Au sample was also confirmed as metallic gold by examining the Au lattice spacing by high-resolution TEM [*d* = 0.236 nm, (111) plane of Au crystal (PDF 99–0056)] and by SAED (*d* = 0.234 nm, see Figs. S13–S14). These measurements prove the capability of the setup to allow detailed characterization of identical samples by X-ray and electron microscopy, giving access to multiple sample length scales and spatial resolutions down to the sub-nanometre level.

### 3.3. Limited-rotational-angle 3D ptychography of hierarchical macroporous zeolite using the 3G cell   

**Figure 7:**
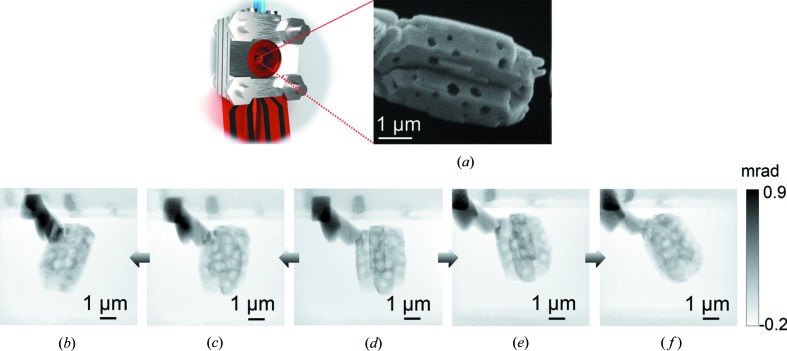
Tilting-angle tests using micro/macroporous zeolite as a case study: (*a*) illustration of the 3G cell with the corresponding SEM image of the zeolite crystal on a MEMS chip, and ptychography images of the specimen at tilting angles with respect to the incident beam: (*b*) 35°, (*c*) 15°, (*d*) 0°, (*e*) −15°, and (*f*) −35°. Dark spots in phase maps indicate areas rich in Si/Al. Pt glue is visible as the dark region in the upper left of each image.

The 3G cell used in this case study matches all the experimental capabilities of the 2G cell described previously. However, the 3G cell was designed to furthermore permit *in situ* imaging under a range of tilting angles, therefore unlocking *in situ* PXCT in a closed cell as a viable characterization technique. To demonstrate the potential of the 3G cell to perform PXCT firstly under *ex situ* conditions, a ptychographic projection series at various tilting angles was acquired from a single crystal of hierarchically porous zeolite. Consisting of 2–4 µm crystals enclosing micropores (<2 nm) along with interior voids of several hundred nanometres, the macroporous zeolite therefore functions as a good test sample for 3D imaging, with strongly identifiable interior features (Machoke *et al.*, 2015 ▸; Schwieger *et al.*, 2016 ▸; Przybilla *et al.*, 2018 ▸). It should be noted that the zeolite micropores present (<2 nm) are challenging to observe except potentially with high-resolution electron microscopy, which was not performed on the specific sample used here. PXCT measurements were performed at ambient temperature and in a He gas environment with the sample placed in the fully assembled 3G cell. Fig. 7 ▸(*d*) shows the ptychographic image taken at 0° tilting angle, *i.e.* with the cell in its normal position perpendicular to the incident beam. The macroporous structure of the zeolite can clearly be observed as a series of spherical voids within the catalyst grain. The permitted angular range was eventually found to be approximately ±35° with the cell fully assembled, as indicated by the image series shown in Fig. 7 ▸. Beyond these, the X-ray beam was partially blocked by the cell body, greatly compromising the quality of the reconstructed images. Notably, by removing the chip from the cell body, rotational angles of ±65° could be reached. While images were acquired at ambient temperature, this was not due to the limited functionality of the cell but rather due to the high thermal stability of the zeolite material and the lack of any obvious thermal annealing process like for np-Au. The zeolite sample served primarily as a test object, but was measured within the assembled 3G cell, which is known to have identical control over temperature and gas conditions as the 2G cell presented earlier. The zeolite serves therefore as a good proof-of-concept study for combining PXCT and *in situ* measurements on other systems in the future, such as *in situ* drying, annealing or chemical reactions. The acquisition of partial data due to geometric limitations draws comparison with both electron tomography, where missing wedges are unavoidable (Arslan *et al.*, 2006 ▸), and lamino­graphy, where the sample is filtered out of the plane of the X-ray beam and rotated non-perpendicularly with respect to the beam (Odstrcil *et al.*, 2018 ▸). However, in both cases, *in situ* implementation of such methods can be challenging under a controlled temperature and gas environment, while the geometry of the 3G cell has more in common with electron tomography than lamino­graphy.

**Figure 8:**
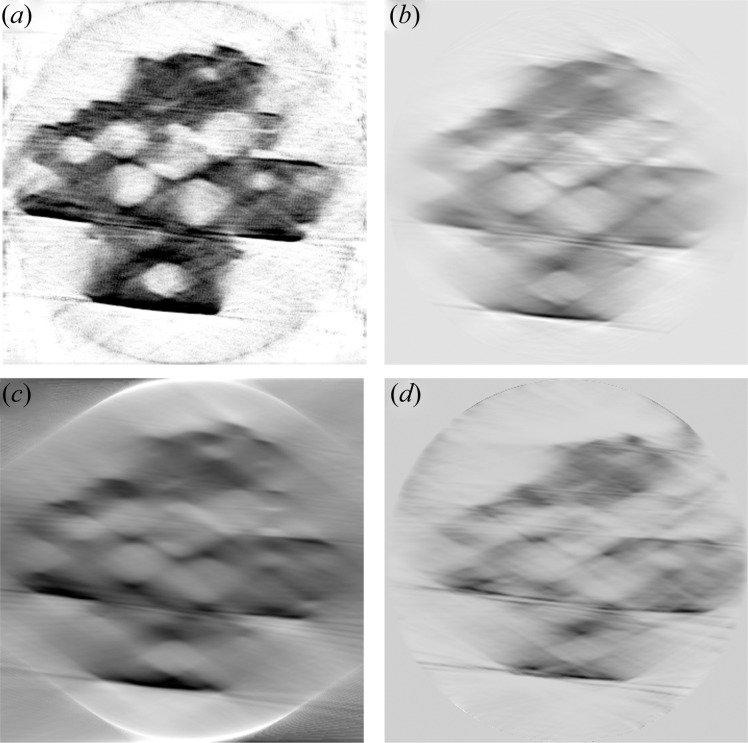
Single slice following tomographic reconstruction of the hierarchical zeolite sample from limited-angle data by: (*a*) an in-house deep-learning method (under development at P06), (*b*) SART from the *scikit-image* Python package; (*c*) SIRT from the *TomoJ* plugin of *FIJI* software and (*d*) MLEM using in-house code.

Despite the limited rotational range and relatively low number of projections acquired leading to a large missing wedge, it was possible to perform rudimentary tomographic reconstruction of the data. The results of several reconstruction methods are shown in Fig. 8 ▸. As expected from the suboptimal data-acquisition parameters and the missing wedge, typical reconstruction methods such as SART, SIRT and MLEM, produce significant artifacts such as ‘cats eye’ broadening of the roughly spherical zeolite pores, and significant blurring of edge features (Kupsch *et al.*, 2016 ▸). In an attempt to improve the reconstruction quality and mitigate missing-wedge effects, a deep-learning reconstruction technique currently in development at PETRA III (DESY) for handling such data was employed. As shown in Fig. 8 ▸(*a*), this significantly improved the appearance of the zeolite data, where the pores, which are known to be close to spherical (Fig. 7 ▸), also appear as spherical pores. While the application of this technique is still at an early stage, there are in any case several opportunities for improving the data-acquisition range and therefore the quality of the reconstructed data which can be obtained by any of the above reconstruction techniques. Firstly, in the case that a contained sample environment is not necessary throughout an entire experiment, the sample can readily be treated under certain conditions of temperature or gas, then simply removed from the cell at regular intervals to perform tomography scans. This procedure could be repeated to obtain an image series during thermal treatment covering ±65°, which nearly doubles the measurable angular range and more than halves the size of the missing wedge compared with a rotational series inside the cell. Notably, an angular range of ±65° is similar to the missing-wedge data routinely recorded using electron tomography (Zečević *et al.*, 2013 ▸; Midgley & Dunin-Borkowski, 2009 ▸). Secondly, if maintaining an *in situ* environment is essential to the experiment (*e.g.* because of sample instability in air or water), the ±35° series acquired during temperature or gas treatment could be supplemented with a more detailed ±65° scan both before and after the experiment. In the latter case, the acquisition of additional angles may supplement the deep-learning method by allowing the training of data reconstruction for the more limited-angle scans. Improved data quality could also naturally be obtained by an increased number of projections, *i.e.* choosing a more appropriate level of angular sampling on the non-missing-wedge, which was undersampled in the present work. In any case, the extended time required for appropriate sampling over a suitable angular range should be carefully considered for potential *in situ* experiments, where PXCT series should be acquired during long sequences of different experimental conditions. Furthermore, while not optimal for technical catalysts where the sample is ideally studied with a minimum of preparation to preserve the structure and function, reducing the sample thickness may relax somewhat the angular requirements for tomographic reconstruction (Madejski *et al.*, 2018 ▸). In summary, this proof-of-concept acquisition of a missing-wedge dataset complete with preliminary reconstruction indicates the potential of the 3G cell for collecting full 3D data series, although further development of acquisition parameters and reconstruction methods is certainly a priority. In future, by utilizing the full function of the 3D cell, the ability to record even limited 3D data series under fully *in situ* conditions should prove to be interesting for the further development of synchrotron PXCT. The feasibility of such experiments is indicated here using the 3G cell apparatus.

## 4. Discussion   

While the 2G and 3G cells described here have essentially finished commissioning and are ready to be employed for general users of beamline P06 and PETRA III, several improvements and future advances are nevertheless anticipated. Potential improvements to the cell design could focus on reducing the physical profile and mass even further, therefore leading to improved sample stability and minimizing the harmful effects of mechanical vibrations on spatial resolution. In any case, sample stability has been shown to be marginally less crucial for ptychography than for analogous measurements using STXM for example, since ptychography can readily operate with a less-focused beam or with the sample out of the focal position, while still obtaining superior spatial resolution in the reconstructed data (Pfeiffer, 2018 ▸; Schroer *et al.*, 2017 ▸). For the 3G cell specifically, increasing the possible rotational angles by thinning the cell-body design to increase the solid angle, or using alternative MEMS chips designed specifically for tomography, may be readily achievable in the near future. To improve the range of experimental conditions, alternative window materials to thin Kapton could be explored to allow potential studies at elevated pressures greater than 100 kPa. Finally, there is significant room for development of 3D PXCT under *in situ* conditions, particularly concerning data analysis. It should be noted that reconstruction of 3D tomographic data with limited rotational angles may be challenging and prone to artifacts and errors depending on the reconstruction algorithm, although missing-wedge measurements are typically acquired as standard using electron tomography. Data reconstruction could also be assisted by complementary TEM tomography, provided the sample dimensions allow for both TEM tomography and PXCT on the same region of interest. As the 2G and 3G cells are developed as an enabling technology for general users of beamline P06 and PETRA III, considering the range of potential users and sample types investigated at nanoprobe beamlines, it is feasible to assume that not all possible functions of the cell may necessarily be needed simultaneously. In any case, the potential of the 3G cell for performing PXCT under *in situ* conditions, even with currently limited angular range, is a remarkable advance on currently available sample environments, and may find widespread use beyond the field of catalysis. Finally, it is also important to note the imminent arrival or planning of fourth-generation and/or diffraction-limited storage rings, such as ESRF-EBS, PETRA IV or SLS 2.0 (Schroer *et al.*, 2018 ▸). The improved beam properties are predicted to strongly benefit ptychography, particularly through the increased coherent flux available. Therefore, the time is right to develop and commission dedicated sample environments to take full advantage of improvements in imaging speed, spatial resolution and coherent flux, and to fully exploit these new capabilities for the catalysis and materials science community.

## 5. Conclusions   

Two new modular ‘nanoreactors’ for complementary imaging at synchrotron radiation sources and in electron microscopes have been designed and commissioned at the hard X-ray nanoprobe endstation at beamline P06 of the PETRA III synchrotron radiation source. The cells offer numerous advantages over previously available sample environments. *In situ* XRP studies of thermal annealing processes under gas flow on monolithic np-Au were successfully conducted using an accurate and easily controlled heating and gas-flow setup. For the implementation of thermal annealing processes, the sample deposition, accurate control and ease of use of the temperature-controller setup are of high importance. Complementary TEM measurements on the same MEMS chip were also demonstrated, showing the ability to assess multiple hierarchical length scales of the same sample. Hierarchical zeolite samples were used to demonstrate the tilting capability of the cells for potential ptychographic tomographic studies on catalysts and functional materials, either: (i) under *in situ* conditions or (ii) through sequential treatment under reaction conditions, followed by imaging outside of the cell. However, the limitations of collecting and reconstructing tomography data with less than 180° angular range should be carefully considered. Other potential studies may include: (i) elemental analysis through fluorescence measurements at the synchrotron or using complementary TEM-EDX; (ii) crystallinity analysis through large-angle XRD at the synchrotron and/or SAED for electron microscopy; and (iii) oxidation-state analysis through tuning incident X-ray energy around an absorption edge at the synchrotron (resonant ptychographic imaging), or by electron energy-loss spectroscopy for electron microscopy. A variety of applications ranging from gas sensing, gas storage, redox reactions and thermal treatments, can benefit from use of these cells, which will presently be made available for general users of beamline P06 and PETRA III.

## Supplementary Material

Supporting information. DOI: 10.1107/S160057751900660X/pp5141sup1.pdf

## Figures and Tables

**Table 1:** Experimental parameters during XRP studies at P06 beamline of PETRA III

Sample type	np-Au	np-Au	Zeolite
Energy (keV)	9.0	9.0	9.0
Sample-to-detector distance (mm)	2310	2310	3470
Sample focus distance (mm)	In focus	In focus	0.6
Exposure time (ms)	10	5	500
Scan time per image (min)	3–4	4–5	5–6
Beam size on the sample (nm)	60	60	2000†
No. of scan points	125 × 125 = 15625	200 × 200 = 40000	12 × 12 = 144
Scan step size (nm)	40	100	333
Scan type and dimensions (µm)	Continuous, 5 × 5	Continuous, 20 × 20	Grid jitter mode, 4 × 4
Optics	FZP‡, 125 µm aperture, 70 nm outermost-zone width, 30 µm pinhole, 60 µm central stop, 63.5 mm focal length, 1 mrad beam divergence		

† Out of focus.

‡ Fresnel zone plates.
