# Supplementary material for: A versatile nanoreactor for complementary in situ X-ray and electron microscopy studies in catalysis and materials science
Source: J Synchrotron Radiat. 2019 Aug 27;26(Pt 5):1769–81. doi: 10.1107/S160057751900660X (PMC6732905; doi:10.1107/S160057751900660X)
Supplement: Supplementary file 1 [file s-26-01769-sup1.pdf]

JOURNAL OF SYNCHROTRON RADIATION

Volume 26 (2019)

Supporting information for article:

A versatile nanoreactor for complementary *in situ* X-ray and electron microscopy studies in catalysis and materials science

Yakub Fam, Thomas L. Sheppard, Johannes Becher, Dennis Scherhaufer, Heinz Lambach, Satishkumar Kulkarni, Thomas F. Keller, Arne Wittstock, Felix Wittwer, Martin Seyrich, Dennis Brueckner, Maik Kahnt, Xiaogang Yang, Andreas Schropp, Andreas Stierle, Christian G. Schroer and Jan-Dierk Grunwaldt

S1. Gas flow simulation of 2G/3G cells

In ANSYS AIM (Student Version, ANSYS, Inc.), Fluid Flow template module was used to simulate the gas flow in the 2G and 3G cell. Specific parameters were kept as default unless specifically mentioned. The geometry was generated by the integrated Space Claim Direct Modeler (SDCM) and "Define mesh manually" was chosen. In Fluid-specific options, "Swirling flow" was ticked to enable any significant rotating or twisting movement of the gas. The simulation focus was set as "Steady-state fluid flow". Number of fluid regions was selected to be "1", i.e. Air as the Material Sample, and "Compressible flow (ideal gas)" was ticked as an additional flow physics. For the meshing process settings, the resolution was set to 4, "Fluid flow or fluid-solid heat transfer" was selected for Engineering intent, and the refinement mechanism of "Curvature and proximity" was used. In Mesh controls, the element shape was set to "Automatic", where it meshed sweepable bodies with hexahedrons and provided a tetrahedral mesh on bodies that were not sweepable. The number of generated nodes was 46556 for 2G cell and 95272 for 3G cell, respectively.

For the Physics parameters, the whole SDCM-generated volume was specified as the location of Physics Regions. "Laminar" flow model was selected as the Flow Model since a Reynolds number (Re) of 9 was estimated according to: $Re = \frac{\rho * v * D_H}{\mu}$, where ρ is the mass density of air (1.1839 kg m^{-3} at T = 298 K), υ is the mean velocity of air (0.236 m s^{-1} in this case from a volumetric flow of 1 ml min^{-1}), D_H is the hydraulic diameter of the inlet/outlet channel (0.3 mm), and μ is the dynamic viscosity of air (18.6 μPa s). The Inlet/Outlet Boundary was located on either one cross-section face of the long cylindrical channels with "Mixed" subsonic-supersonic regime and Gauge static pressure of 0 Pa. Additional parameter settings were for the inlet with gas Velocity magnitude of 0.236 m s^{-1} and Temperature of 298 K. The rest of the faces on the SDCM-generated volume was determined as the Wall Boundary. The Solver Options was left as default, where the solution would converge with Residual Tolerance of 1E-05 and Maximum iteration number of 1000; Solution advancement exponent was kept 0 under Calculation Control; and "Least squares cell-based" Gradient method was used for Discretization Control with "Second-order upwind" Advection and "Automatic" Pressure scheme. As the results, the convergence criteria were met after 45 iterations with residual tolerances up to 9.69E-06 for 2G cell, and 773 iterations with residual tolerances up to 2.15E-07 for 3G cell. The fluid flow profiles of the 2G and 3G cell are displayed in Figure 3 of the manuscript.

S2. Sample preparation of np-Au series via FIB

The description below (Figure S1-S4) only applies to the preparation of np-Au samples. Each original sample was a solid pellet, a piece of which was deposited on a SEM stub and then on a lift-out grid, which acted as an intermediate stage to the final sample holder, i.e. MEMS chip (Figure S1). All sample preparation steps were performed in a dual-beam focused ion beam. For details in each stage after mounting the stub, the piece was cut again and shaped into a smaller block / trapezoidal prism (Figure S2). The block was then transferred onto the lift-out grid, where a smaller wedge shape was eventually obtained (Figure S3). Last, the final transfer onto the MEMS-chip was made on the spiral center area of the chip and glued with Pt on the corner to stabilize the position (Figure S4).

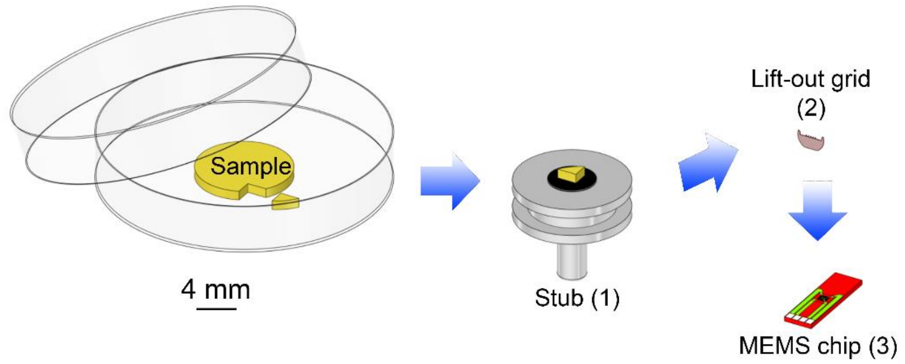

Figure S1 Overview of np-Au sample preparation on the MEMS chip via FIB.

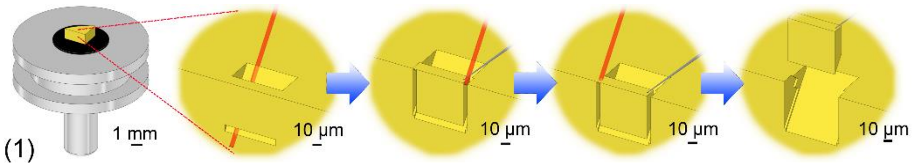

Figure S2 Initial np-Au extraction on a SEM stub to make a block for preparing several samples.

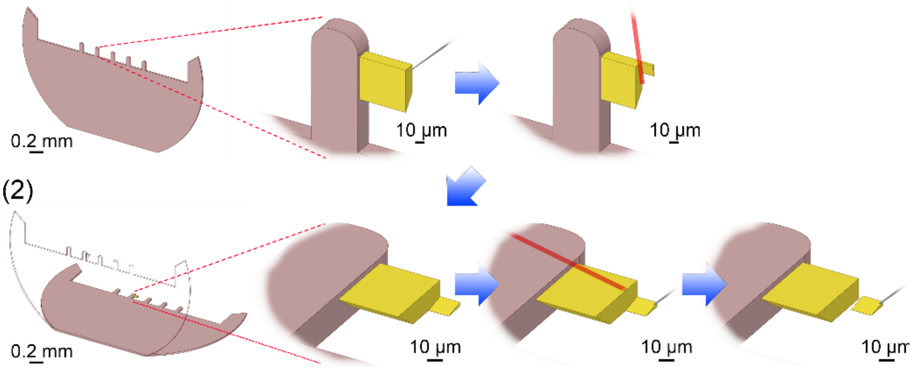

Figure S3 Final cut after transfer to intermediate lift-out grid and thinning of block into a wedge.

(3)

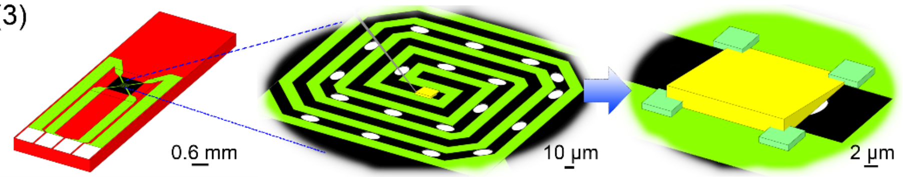

Figure S4 Transfer and deposition of the wedge on the MEMS chip.

S3. Temperature stability of the heating interface system

Since the MEMS chips and parts of heating interface system are commercially available and tested, the temperature readout and control process proved to be robust as shown in Figure S5. Excellent stability can be observed in the step functions between heating points, where fluctuations of <1 K were observed during temperature adjustment.

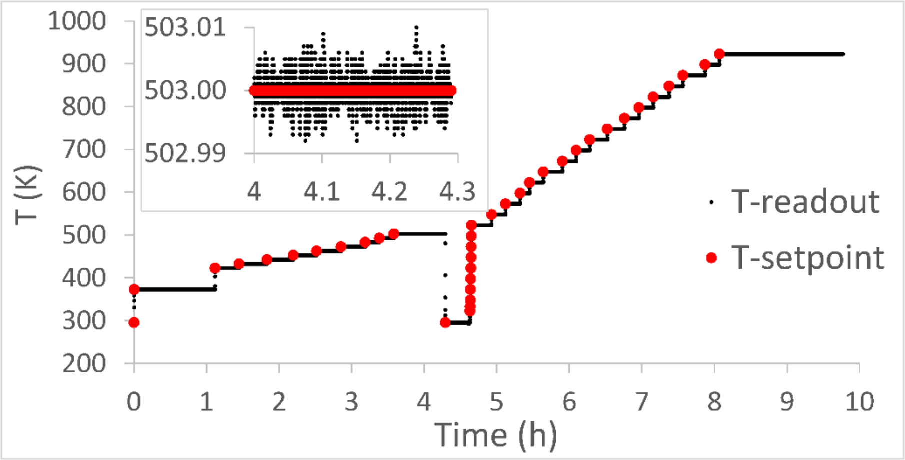

Figure S5 Plot of temperature readout vs time via the integrated temperature control system, recorded during thermal annealing study of nanoporous gold. The valley between the fourth and fifth hour of the measurement resulted from pausing the experiment due to loss of beam.

S4. Determination of the spatial resolution

To estimate the spatial resolution of the ptychograms obtained, a Fourier Ring Correlation (FRC) analysis was performed (Van Heel & Schatz, 2005), depicted in Figure S6 for colloidal Au, Figure S7 for nanoporous gold, and Figure S8 for the hierarchical zeolite particle. The intersection of the FRC datapoint with the 1 -bit threshold curve provides an estimation of the spatial resolution in nm averaged over the whole image and all directions.

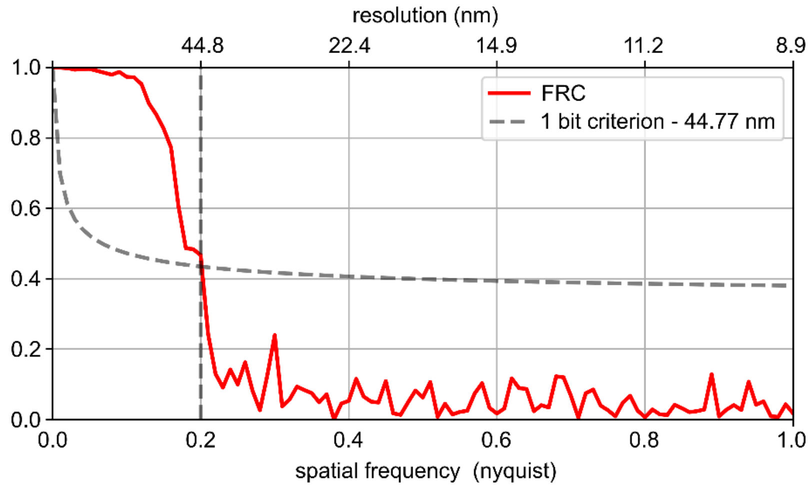

Figure S6 FRC of the ptychogram of colloidal Au nanoparticles.

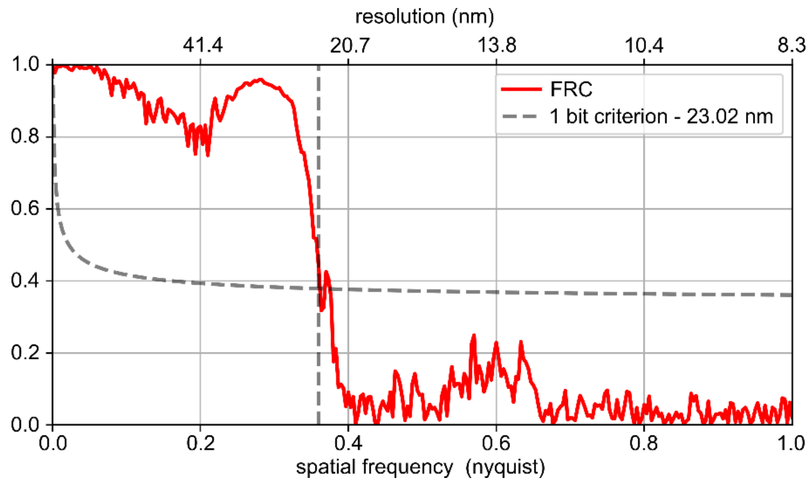

Figure S7 FRC of the ptychogram of np-Au sample.

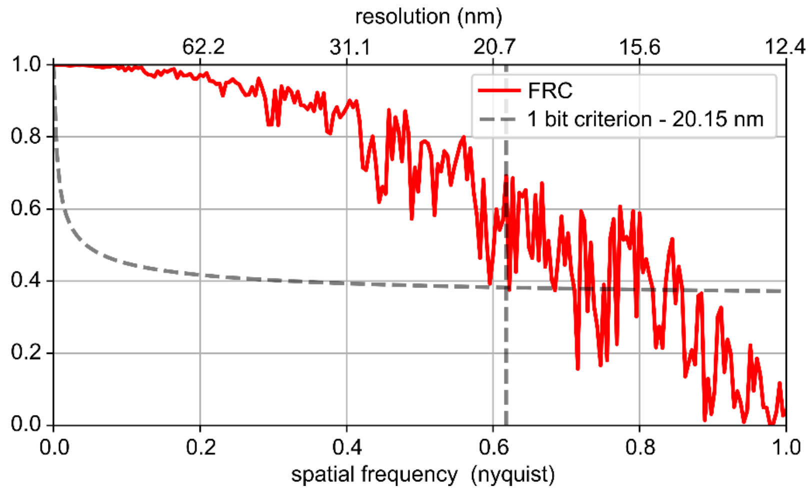

Figure S8 FRC of the ptychogram of zeolite.

In addition to FRC, the resolution of the images was also estimated with a sharp edge fitting as shown in Figure S9. This was performed by drawing a line profile across a sharp contrast feature and counting the number of pixels through which the line passes. It should be noted that this method relies on the presence of sharp features, and is therefore dependent on the specific sample and image contrast. In general, lower estimations of resolution can be expected than for FRC. In particular, since the colloidal gold and zeolite could be clearly measured against the background, a sharper contrast gradient was observed. On the other hand, the np-Au sample is significantly thicker, while the void measured probably does not penetrate through the entire sample.

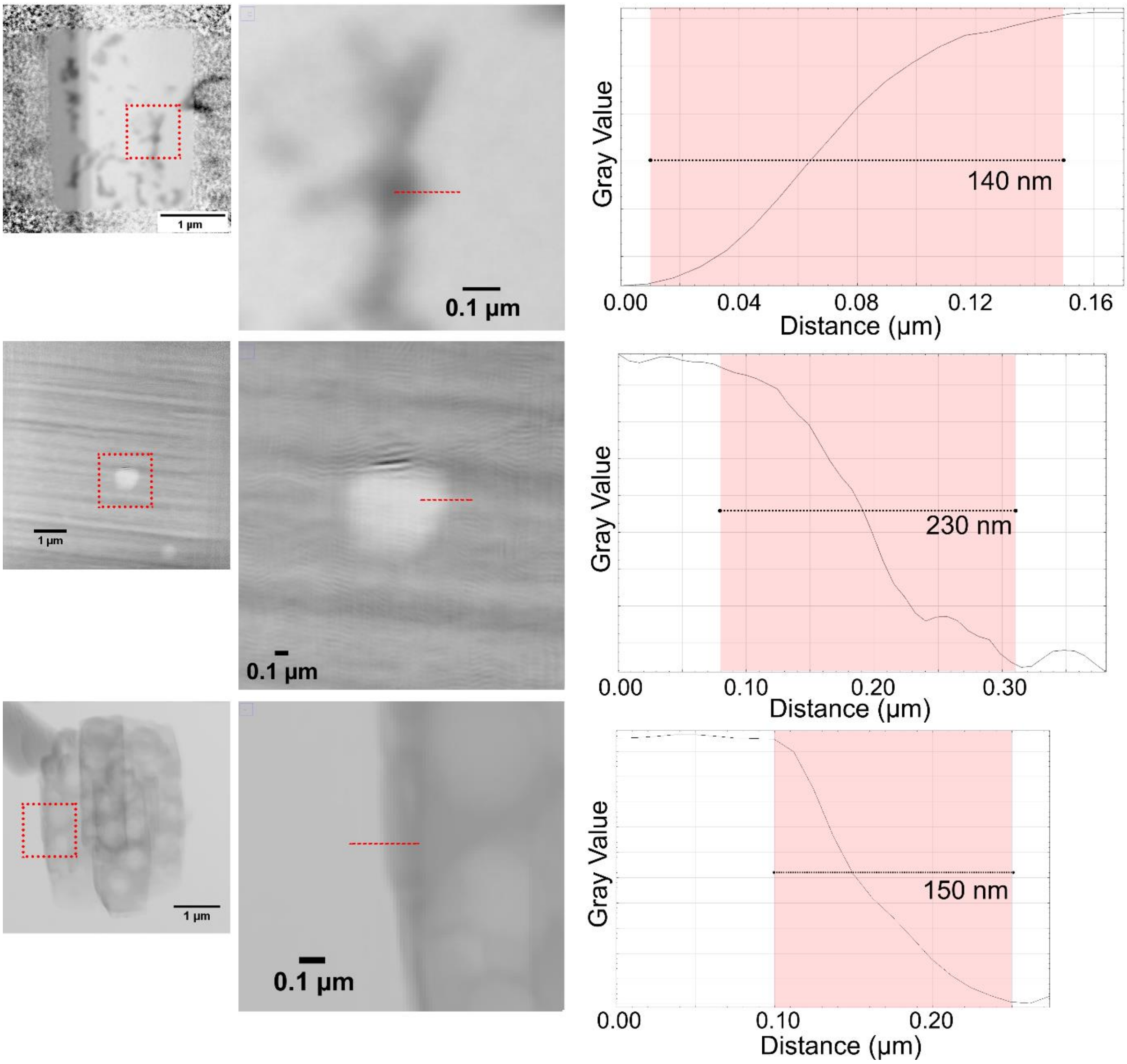

Figure S9 Estimation of spatial resolution via 'Edge Detection' method in FIJI software. The spatial resolution is indicated by intensity difference near the edge area highlighted in red from the graphs. Top: colloidal Au nanoparticles, middle: np-Au, bottom: hierarchical zeolite.

S5. *In situ* sintering of Au colloids – early cell prototypes using 2G cell

A solution of colloidal Au nanoparticles stabilized in citrate (Sigma-Aldrich Chemie GmbH, Taufkirchen, DE) with ~50 nm uniform particle size were applied directly to the sample area of the MEMS chips and the solvent left to evaporate for one hour under flowing air. The experiment using colloidal Au nanoparticle sample was performed at the nanofocus endstation of beamline P06 at the PETRA III synchrotron radiation source (Hamburg, Germany) (Schroer et al., 2017), using the energy of 15 keV, nano-focusing refractive X-ray lenses as the optics, and EIGER X 4M detector. The sample was positioned in the beam focus and located at 4.16 m from the detector. Moreover, the sample was scanned under 2-6 min per image with a field of view of 2.5 x 2.5 μm^2, exposure time of 50 ms, 500 scanpoints, and scanning step size of 100 nm. The algorithm used for ptychographic reconstruction was based on the (e)PIE algorithm. Cropping the diffraction patterns to 256×256 pixels led to a pixel size of 8.9 nm in the reconstructed images. To estimate the spatial resolution, a Fourier ring correlation (FRC) analysis was performed.

During measurement of the Au colloid sample, XRF signal was collected with a single channel Vortex silicon drift detector at 50 nm intervals with a collection time of 50 ms/pixel. The detector was placed on a moveable table and was located at an angle approximately 72 ° to the incident beam. It should be noted that the cell body blocks positioning of the detector ideally perpendicular to the incident beam. An XRF scan in high flux mode was recorded by scanning the sample over a field of view of 5x5 and 15×15 μm^2 with a step size of 50 nm. At each scanning position an XRF spectrum was collected for an exposure time of 2 s. The XRF spectra were fitted by AXIL software for the analysis of line intensities of the elements.

Colloidal Au particles with a size of 50 nm were utilized to confirm the applicability and feasibility of the 2G cell for *in situ* XRP and XRF. For this study, an early prototype of the 2G cell was used. This prototype consisted of the same physical cell profile shown in Figure 2 including electrical contacts, but without the gas tight O-ring and window assembly. Experiments were therefore performed only at elevated temperature up to 773 K but with ambient gas conditions. The ptychography images (Figure S10 – top row) imply that the spatial resolution should be at least 50 nm as colloidal Au can be clearly resolved, where high local concentration of colloidal Au is indicated by the darker intensity. Using FRC, the spatial resolution was in fact estimated to be 45 nm as shown in Figure S6. However, the Au fluorescence maps (Figure S10 – bottom row) are more suitable for locating colloidal Au, with increased local concentration leading to higher fluorescence counts. The Au colloids showed a distribution of larger connected ligaments, likely from clustered bunches of colloids, together with a homogeneous distribution of smaller particles, particularly visible in the upper middle of Figure S10a-b. During temperature treatment, the colloidal Au underwent several modifications including aggregation and/or recombination of individual particles into larger clusters. This was evidenced by the contraction of ligament shapes into spherical bodies which began already at 423 K, together with

the apparent disappearance of smaller particles in close vicinity to the more densely concentrated ones. These observations are typically expected for unsupported particles at elevated temperatures, at which the organic surfactant likely decomposes leading to aggregation. The complete disappearance of many smaller Au particles was also observed particularly at 773 K, which was somewhat unexpected since particle migration may not be expected to proceed across several microns. However, this could be due to the unstable deposition of unsupported particles directly on the Si_3N_4 window of the MEMS-chip, so the thermal effect may have caused them to significantly drift or fall away from the chip. As noted above, instead of the Digiheater temperature control unit, a variable voltage power supply and an IR thermographic camera were used as in previous work (Baier, Damsgaard *et al.*, 2016). A typical IR thermography image captured from the cell during operation is shown in Figure S11. In this case, wires connected to the power supply were soldered directly to the power in/out contact of the printed circuit. As such, accurate control of the sample temperature was not guaranteed during the experiment. As noted previously, a sudden variation in local gas composition or local chemical reactions on the chip may cause significant temperature spikes, as observed in previous work (Baier, Damsgaard, *et al.*, 2016). This might also contribute to the sudden disappearance of colloidal Au at 773 K. This early case study highlights the necessity of a stable and dependable temperature control system, which was later integrated into both the 2G and 3G setups detailed in Figure 1 and Figure 2.

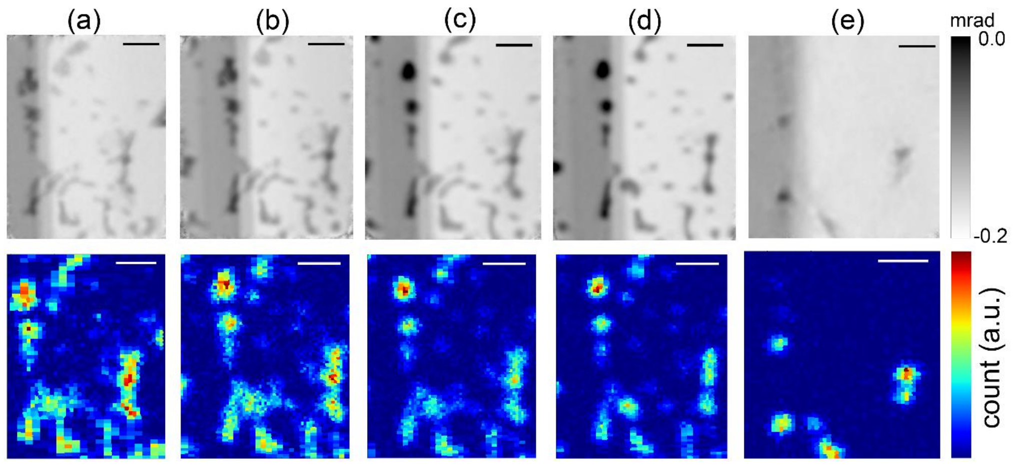

Figure S10 Ptychography images (top) and Au fluorescence maps (bottom) during thermal annealing of colloidal Au: (a) room temperature (293 K), (b) 423 K, (c) 573 K, (d) 723 K, and (e) 773 K. Dark spots in phase maps and high count zones in fluorescence maps indicate areas most rich in Au. Scale bars = 500 nm.

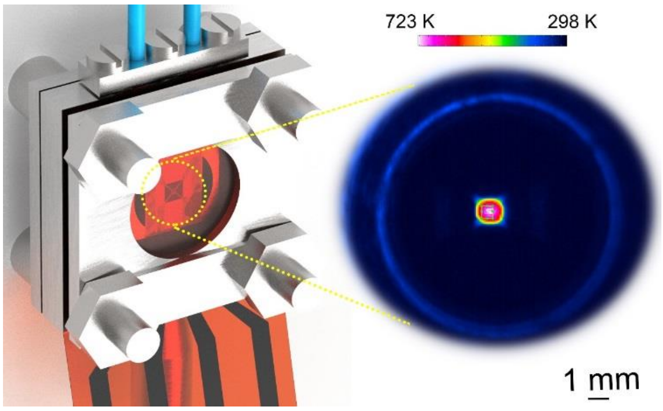

Figure S11 Depiction of the 2G cell (left) and the temperature profile on the MEMS chip recorded by IR thermography (right). The image was recorded inside the assembled prototype 2G cell during thermal annealing of the colloidal Au sample. Heating was performed via Joule heating principle on only 2 out of 4 pads located on MEMS chips, so the electrical currents could be converted into heat.

S6. Electron microscopy analysis of nanoporous gold

Additional high resolution TEM images, EDX analysis and selected area electron diffraction data were recorded on the nanoporous gold sample described in the study. Data was recorded on the sample deposited on a MEMS chip and placed within a Wildfire sample holder (DENSsolutions, Delft, NL), on a Titan 80-300 (FEI) microscope operated at an acceleration voltage of 300 kV in STEM and TEM mode, at the Institute of Nanotechnology (INT) at Karlsruhe Institute of Technology.

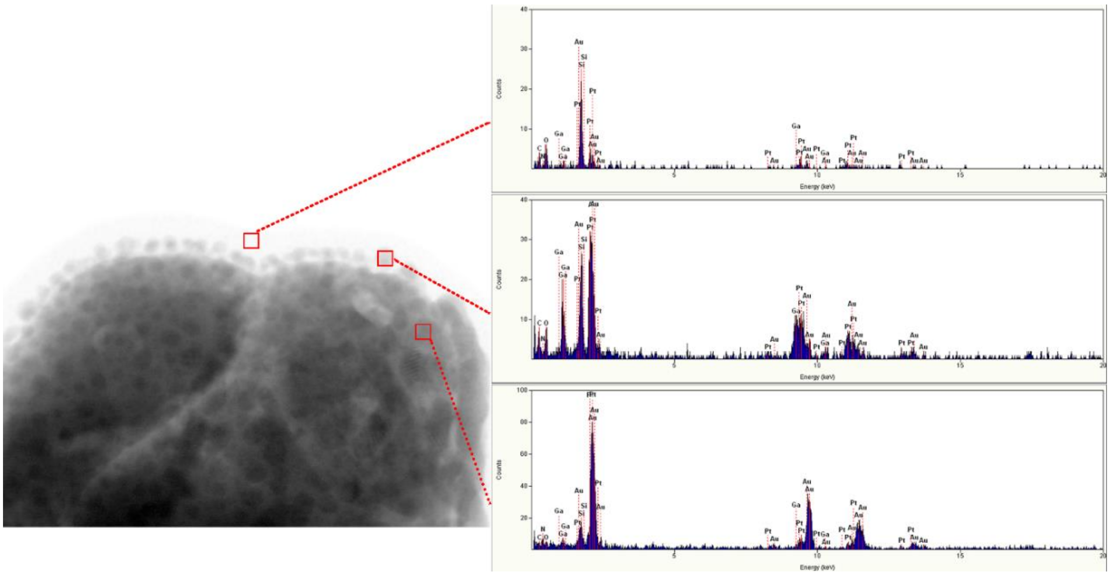

Figure S12 TEM-EDX data recorded on the nanoporous gold sample used for *in situ* ptychography. Analysis shows the presence of minor Si and Pt contaminants close to the Au ligament edges.

Table S1 Quantification of EDX spectra in Figure S12.

Element	Atomic %		
	Area 1	Area 2	Area 3
C	6.2	29.6	29.1
N	6.9	0.0	3.9
O	5.2	13.1	14.2
Si	21.3	32.0	52.7
Ga	0.1	12.1	0.0
Pt	0.0	10.6	0.0
Au	60.2	2.6	0.0

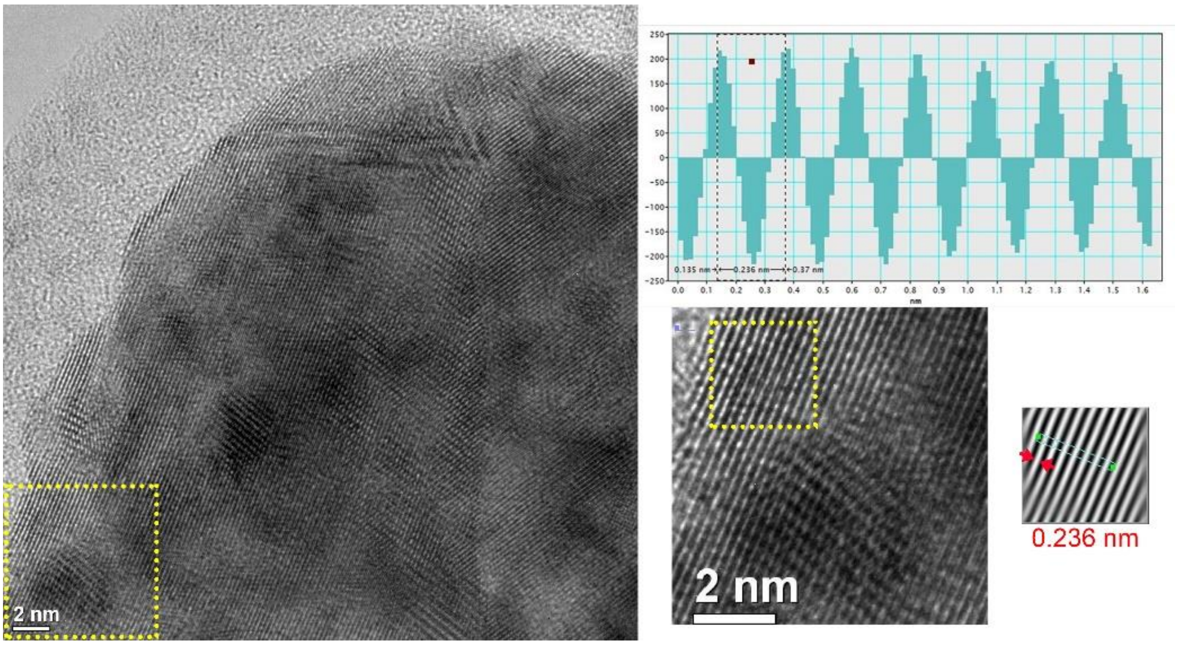

Figure S13 Lattice spacing measurement of fresh np-Au sample (highlighted area) via bright-field high resolution TEM, indicating d = 0.236 nm, belonging to (1 1 1) plane of Au metal (PDF 99-0056).

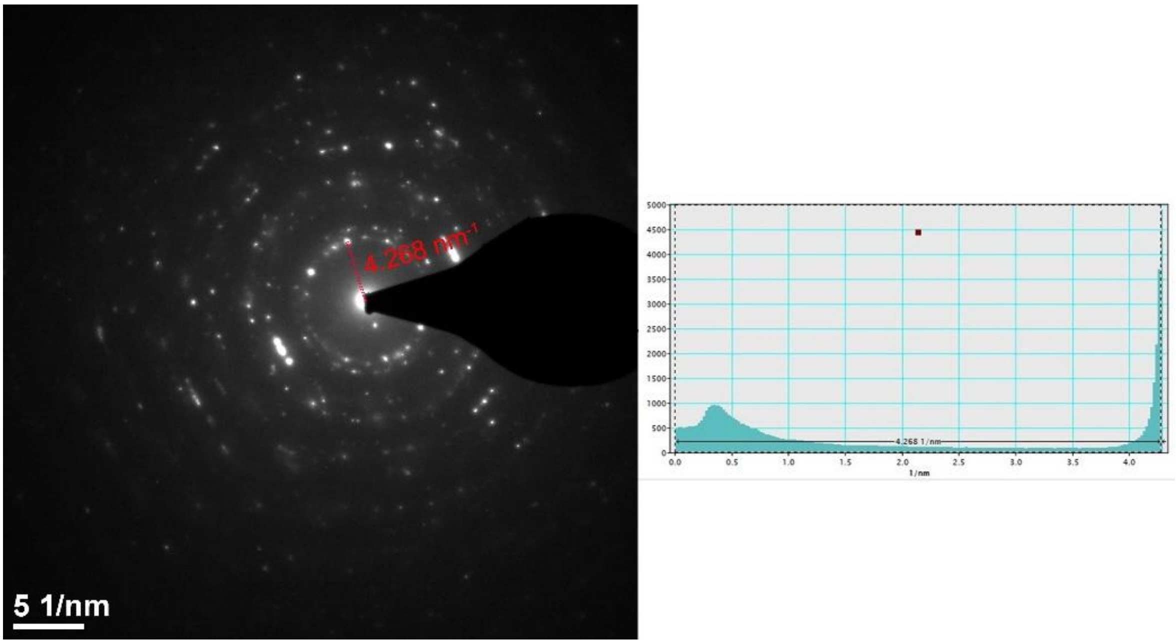

Figure S14 Lattice spacing measurement of fresh np-Au sample (between indicated points) via selected area electron diffraction, indicating d = 0.234 nm, belonging to (1 1 1) plane of Au metal (PDF 99-0056).
